# Correction: Direct access to the graphene–metal interface using Raman spectroscopy to study the origin of contact resistance in operational devices

**DOI:** 10.1039/d6na90080a

**Published:** 2026-10-27

**Authors:** Alessandro Kovtun, Leonardo Martini, Piera Maccagnani

**Affiliations:** a Consiglio Nazionale delle Ricerche, Istituto per la Sintesi Organica e la Fotoreattività, (CNR-ISOF) 40129 Bologna Italy alessandro.kovtun@cnr.it; b Dipartimento di Scienze Fisiche, Informatiche e Matematiche (FIM), Università di Modena e Reggio Emilia 41125 Modena Italy; c Consiglio Nazionale delle Ricerche, Istituto per lo Studio dei Materiali Nanostrutturati, (CNR-ISMN) 40129 Bologna Italy piera.maccagnani@cnr.it

## Abstract

Correction for ‘Direct access to the graphene–metal interface using Raman spectroscopy to study the origin of contact resistance in operational devices’ by Alessandro Kovtun *et al.*, *Nanoscale Adv.*, 2025, **7**, 7891–7898, https://doi.org/10.1039/d5na00846h.

The authors regret that the Table 1 of the original article contains errors in the contact resistivity values in column 3. The correct version of Table 1 is displayed here.

**Table 1:** Main parameters extracted from the electrical measurements on van der Pauw and TLM structures in graphene, contacted using Cr/Au (20/70 nm). The method used is explained in the SI. The data are averaged over several TLM structures. *R*_SH_ is the sheet resistance of graphene in the region between two metal contacts, *R*_SK_ is the sheet resistance of graphene under the metal contact, *L*_TK_ is the portion of the contact involved in conduction and *ρ*_C_ is the specific contact resistivity associated to the graphene–metal interface

Sample	*R*_SH_ [Ω sq^−1^]	*ρ*_C_ [Ω cm^2^]	*L*_TK_ [µm]	*R*_SK_ [kΩ]
G/a-SiN	810 ± 44	(2.0 ± 0.2) × 10^−4^	1.8 ± 0.3	9.0 ± 1.4
G/SiO_2_	843 ± 90	(1.6 ± 0.7) × 10^−4^	3.1 ± 0.7	1.3 ± 0.5

This correction does not change the conclusions of the original article.

The Royal Society of Chemistry apologises for these errors and any consequent inconvenience to authors and readers.

